# Explainable Agentic Artificial Intelligence in Healthcare: A Scoping Review

**DOI:** 10.3390/bioengineering13050513

**Published:** 2026-04-28

**Authors:** Bernardo G. Collaco, Srinivasagam Prabha, Cesar A. Gomez-Cabello, Syed Ali Haider, Ariana Genovese, Nadia G. Wood, Narayanan Gopala, Raghunath Raman, Erik O. Hester, Antonio Jorge Forte

**Affiliations:** 1 Division of Plastic Surgery, Mayo Clinic, 4500 San Pablo Rd S, Jacksonville, FL 32224, USA; 2 Department of Radiology AI IT, Mayo Clinic, Rochester, MN 55905, USA; 3 Center for Digital Health, Mayo Clinic, Rochester, MN 55905, USA; 4 Department of Artificial Intelligence and Informatics, Mayo Clinic, Jacksonville, FL 32224, USA

**Keywords:** artificial intelligence, agentic AI, explainable AI, explainable agentic AI, healthcare AI, clinical decision support systems

## Abstract

Background: Agentic artificial intelligence (AI) systems, characterized by autonomous goal-directed behavior, multi-step reasoning, task decomposition, and tool use, are increasingly proposed for healthcare applications. However, their autonomy raises concerns regarding transparency, accountability, and human oversight. While explainable AI (XAI) has been widely studied in traditional predictive models, less is known about how explainability is implemented within agentic architectures. Objective: To map the emerging literature on explainable agentic AI (XAAI) in healthcare and characterize the types, scope, and forms of explainability used in these systems. Methods: A scoping review was conducted following PRISMA-ScR guidelines. PubMed, Embase, IEEE Xplore, and ACM Digital Library were searched through November 2025. Eligible studies described healthcare-related agentic AI systems incorporating explicit explainability mechanisms. Data were extracted on system architecture, explainability type (intrinsic, post hoc, hybrid), explanation scope (local, global), explanation form, and reported clinical outcomes. Results: Nine studies met the inclusion criteria. All systems demonstrated core agentic features, including autonomy, task decomposition, and tool integration, often within multi-agent frameworks. Explainability was predominantly intrinsic and workflow-native, typically delivered through textual reasoning traces and example-based grounding in retrieved clinical evidence. Feature-based and global explanations were comparatively rare and largely confined to hybrid architectures. Across domains including radiology, neurology, psychiatry, and biomedical research, XAAI systems were reported to improve performance and interpretability relative to baseline models in the included studies. However, these findings were derived from heterogeneous, predominantly experimental or retrospective studies, and structured human-in-the-loop oversight was infrequently described. Conclusions: Current XAAI systems appear to emphasize process transparency and evidence grounding rather than mechanistic model-level attribution. The available evidence remains limited and heterogeneous, and findings should be interpreted as early trends rather than established characteristics. Further progress will require standardized evaluation frameworks, clearer reporting of oversight mechanisms, and validation in real-world clinical settings to support safe and trustworthy integration of agentic AI into healthcare practice.

## 1. Introduction

The rapid evolution of artificial intelligence (AI) has given rise to a new class of systems commonly referred to as agentic AI, characterized by autonomous, goal-directed behavior, multi-step reasoning, tool use, and, increasingly, multi-agent coordination [1,2]. Unlike conventional large language model (LLM) pipelines or static prompt-based workflows, these systems are designed to plan, decompose, and execute complex tasks through iterative and often self-directed processes. By distributing subtasks across specialized roles and enabling structured reasoning and verification, agentic AI holds promise for addressing the growing complexity of clinical workflows and supporting clinicians in decision-making [3]. As such, agentic AI represents a potentially transformative paradigm for healthcare innovation, offering flexibility, scalability, and contextual reasoning capabilities that extend beyond traditional machine learning approaches [1,4].

Despite this promise, the deployment of agentic AI in healthcare raises critical concerns related to patient safety, accountability, and trust [5]. The autonomous, multi-step nature of agentic systems increases both their expressive power and their potential risk, as errors or unintended behaviors may propagate across reasoning chains or agent interactions, making failures harder to detect and correct. In safety-critical domains such as healthcare, where decisions can directly affect patient outcomes, the ability to understand, audit, and appropriately oversee AI behavior is essential [6]. In this context, it is important to distinguish between the architectural properties of agentic AI and the mechanisms used to make these systems interpretable and governable.

**Figure 1:**
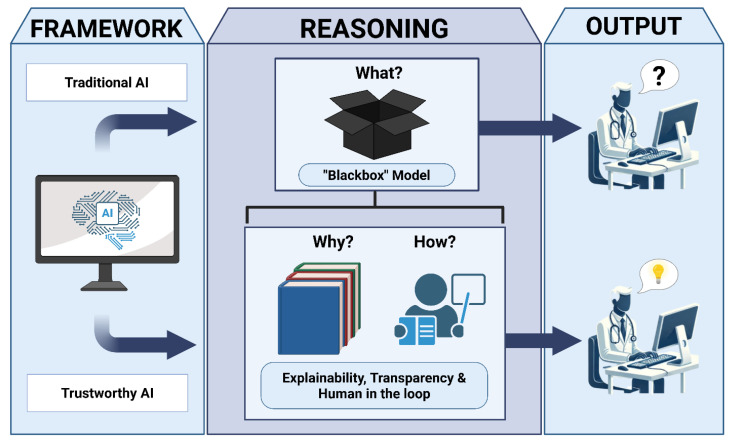
Conceptual transition from traditional “black-box” AI to trustworthy AI through explainability. Created in BioRender. Collaco, B. (2026) https://BioRender.com/3eyye5r (accessed on 12 February 2026).

International and regional guidelines, including the U.S. Food and Drug Administration’s (FDA) framework for AI/ML-based Software as a Medical Device [7], the European Union’s AI Act [8], and the World Health Organization’s guidance on the ethics and governance of AI for health [9], consistently emphasize the importance of transparency, explainability, human oversight, and risk management in healthcare AI (Figure 1). Transparency refers to the visibility of system design and operation, including intermediate reasoning steps, agent interactions, and tool use, whereas explainability refers to mechanisms that provide human-interpretable justifications for specific outputs or decisions [10]. While related, these concepts are not equivalent: agentic AI describes how systems are structured and operate, transparency concerns what aspects of this process are observable, and explainability concerns how outputs are justified to the user. These frameworks underscore that the safe and responsible adoption of agentic AI in clinical settings requires systems to be transparent, interpretable, and controllable, particularly given the well-documented concerns surrounding “black box” models whose internal decision-making processes are opaque to users [11].

Although recent reviews have examined the capabilities, architectures, and clinical applications of agentic AI in healthcare, explainability has been largely treated as a conceptual requirement rather than an operational property of these systems [1,4,12]. There remains limited structured synthesis of how explainability is implemented within agentic workflows, how explanations are generated during multi-step reasoning, how they are presented to users, and how they support transparency, verification, and oversight in practice. This gap is notable because agentic architectures fundamentally shift explainability from static model interpretation toward dynamic, process-level transparency embedded within reasoning and tool use.

In this context, the objective of this scoping review is to map the current literature on explainable agentic AI (XAAI) systems in healthcare and systematically characterize the types, scope, and forms of explainability mechanisms embedded within these systems. We aim to identify how explainability is operationalized within agentic AI frameworks and to examine its role across different clinical and biomedical applications. By synthesizing existing approaches and highlighting gaps in reporting and evaluation, this review seeks to provide a structured overview of the emerging XAAI landscape and to inform future research, development, and governance efforts toward the safe and transparent integration of agentic AI into healthcare practice.

## 2. Materials and Methods

This study was conducted in accordance with the Preferred Reporting Items for Systematic Reviews and Meta-analyses for Scoping Reviews (PRISMA-ScR), with the checklist provided in Supplementary Document S1 [13]. A description of this study’s protocol was registered in the Open Science Framework (OSF) and is available at https://doi.org/10.17605/OSF.IO/8DT7R (accessed on 11 February 2026).

### 2.1. Eligibility Criteria

The review included studies that investigated or described XAAI systems in healthcare or health-related contexts, including clinical decision support systems (CDSSs), diagnostics, treatment planning, patient monitoring, and healthcare operations. To be considered agentic AI, eligible systems were required to demonstrate autonomous, goal-directed execution with explicit multi-step reasoning or task decomposition, often involving tool use or coordinated agent roles [1,5]. In addition, they needed to include explainability or transparency features, such as interpretable models, justification mechanisms, explainable AI (XAI) methods, or user-facing explanations [14]. We included empirical research, simulations, prototype descriptions, system architecture papers, algorithmic evaluations, and qualitative studies involving clinicians or end-users. Both peer-reviewed journal articles and publicly available preprints were considered, with no restriction on publication date or language.

We excluded studies that operate outside healthcare or health-related domains, including those focused on finance, manufacturing, education, transportation, or general AI agents without health applicability. Studies involving non-agentic AI systems, such as static predictive models lacking autonomous behavior, or agentic systems without explainability features, were also excluded. Studies that mention autonomy or explainability but do not provide sufficient methodological, conceptual, or technical detail to evaluate their relevance to explainable agentic AI were not considered.

### 2.2. Study Screening

A comprehensive search was conducted on 24 November 2025, by two independent authors (BC and SP) across four databases: PubMed, Embase, IEEE Xplore, and ACM Digital Library. These databases were deliberately selected to ensure robust coverage of healthcare-related AI research across both biomedical and technical domains. The complete search strategy included a combination of terms related to or similar to “Explainable Agentic AI” and “healthcare,” with appropriate adaptations for each database. ACM Digital Library yielded more than 80,000 results. To ensure screening feasibility and avoid retrieving irrelevant records from non-target fields (e.g., references or full-text indexing), a title and abstract filter was applied. This measure was used to improve search precision, although it may have reduced sensitivity by excluding potentially relevant studies not explicitly indexed in these fields, and did not impose conceptual restrictions on study selection. No additional filters or restrictions (e.g., by publication type, date, subject area, or study design) were applied in ACM or any of the other databases. The full strings across all databases are available in Supplementary Document S2.

Before screening, reviewers discussed and aligned themselves on eligibility criteria to ensure consistent application during study selection. The studies retrieved from each database were imported into EndNote (version 21.3), and duplicated records were removed [15]. The papers were first screened based on title and abstract, and the remaining studies were selected for full-text retrieval. Disagreements, including borderline cases that did not clearly meet or exclude eligibility criteria, were resolved through consensus discussion, with a third reviewer involved when necessary to adjudicate unresolved conflicts (SH). The Robvis tool was used to generate the PRISMA flow chart [16].

### 2.3. Data Extraction and Synthesis

**Figure 2:**
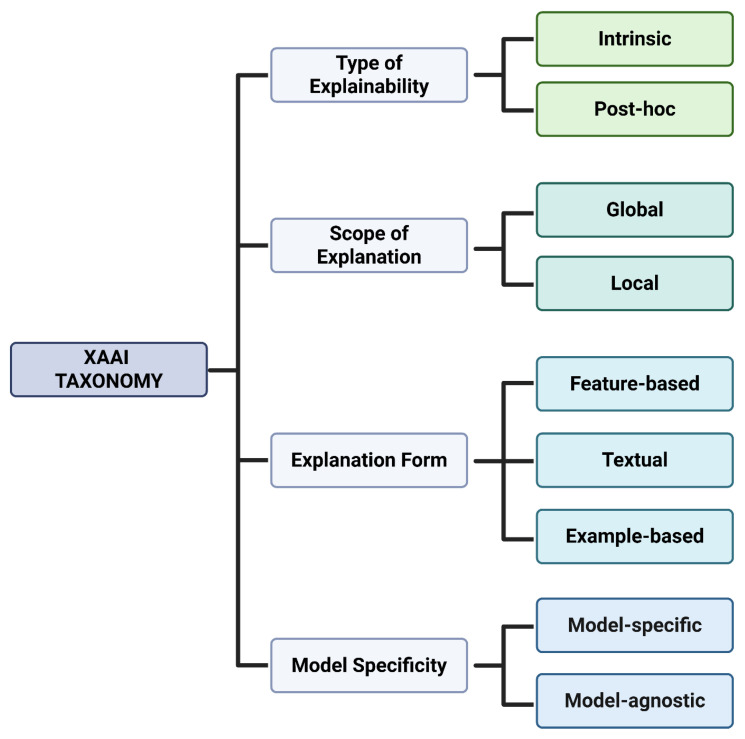
Proposed taxonomy of explainable agentic artificial intelligence (XAAI) in healthcare. Created in BioRender. Collaco, B. (2026) https://BioRender.com/3eyye5r (accessed on 12 February 2026).

For each included study, two independent reviewers (BC and AG) manually extracted bibliographic and methodological characteristics (year of publication, country, and study design) and described the agentic system, including the name of the XAAI, the primary clinical or biomedical task addressed, and relevant details of each architecture. The extracted variables were compared and reconciled to ensure consistency and accuracy. With respect to explainability, we extracted information on the type of explainability (intrinsic, post hoc, or hybrid), the scope of explanation (local, global, or both), which explanation form was used (feature-based, textual, or example-based), specificity (model-specific or agnostic), and the explainable approach employed. In addition, explanations were conceptually interpreted as either process-level (e.g., reasoning traces and workflow transparency) or mechanistic (e.g., feature attribution or model-based explanations) to support consistent interpretation across heterogeneous systems. These dimensions were categorized according to established and previously validated XAI taxonomies [12,17,18], which were adapted to the context of agentic AI systems as a provisional analytic schema to enable consistent classification across heterogeneous implementations. The resulting taxonomy is depicted in Figure 2.

Explainability was categorized as intrinsic when explanations were generated as part of the system’s reasoning or decision-making workflow (e.g., structured deliberation, explicit reasoning steps, rule-based justification, or argumentation procedures), post hoc when explanations were produced after inference without influencing the underlying decision process (e.g., feature attribution, saliency maps, or SHAP/LIME-style methods), and hybrid when both intrinsic and post hoc mechanisms were combined within the same system [12,18]. The scope of explanation was defined as local when explanations pertained to individual decisions, instances, or queries; global when explanations aimed to characterize overall system behavior, decision logic, or generalizable rules; and both when systems supported multiple levels of explanation [17,18]. With respect to specificity, explainability was classified as model- or system-specific when it depended on the structure of the underlying system (as is common for intrinsic approaches), or model-agnostic when explanation methods could be applied across different model types as external explainers, typically in post hoc settings. Finally, explanation form was categorized based on how explanations were presented to users, including textual explanations (e.g., step-by-step rationales, reflective critiques, or narrative justifications), feature- or concept-based explanations (e.g., attribution over input variables or human-interpretable concepts, such as saliency or SHAP-style importance), and example-based explanations, in which justifications were supported using retrieved external information such as clinical guidelines, scientific literature, databases, ontologies, or knowledge-graph paths [12,17,18].

We also extracted reported outcomes related to system performance, interpretability, transparency, and trustworthiness, as described by the study authors. Data extraction was conducted with an emphasis on conceptual consistency rather than implementation detail, ensuring that similar explainability mechanisms were classified under a common category across studies. Extracted data were synthesized descriptively and narratively, with findings summarized in tabular form and complemented by a qualitative synthesis to identify recurring explainability patterns, methodological trends, and gaps in current explainable agentic AI research within healthcare and biomedicine. In addition to predefined extraction variables, inductive observations were made during synthesis to identify recurring themes and gaps in reporting, including aspects not consistently or explicitly described across studies. A formal meta-analysis was not performed due to the substantial heterogeneity of the included studies.

## 3. Results

### 3.1. Study Screening and Selection

**Figure 3:**
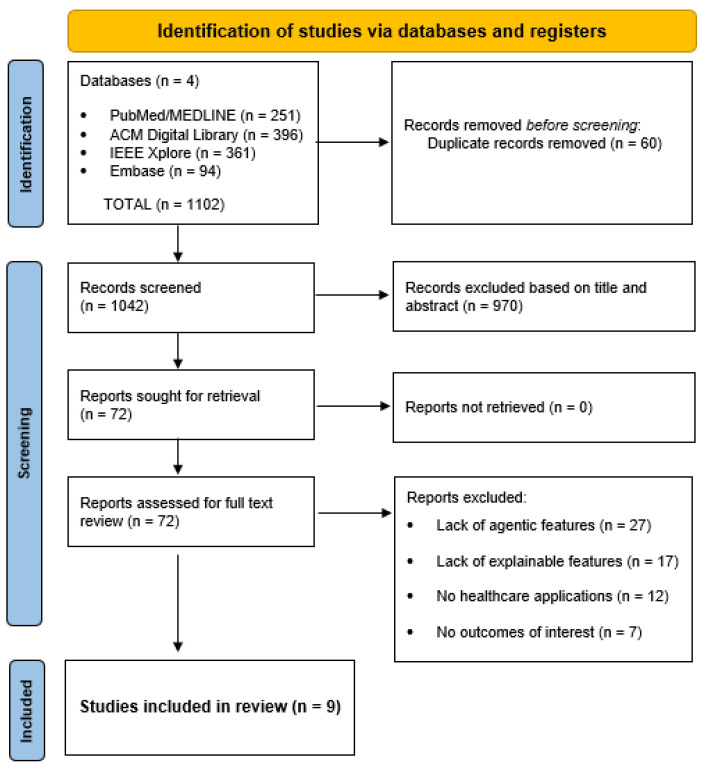
PRISMA flow chart of included studies.

The initial search across the four databases identified 1102 records. After duplicate removal, 1042 studies underwent title and abstract screening, of which 72 were retained for full-text review. Of these, articles were excluded due to a lack of agentic features (n = 27), a lack of explainability features or an unclear approach (n = 17), a lack of healthcare applications (n = 12), or insufficient reported outcomes (n = 7). Ultimately, nine studies met all inclusion criteria and were included in the final synthesis on 15 December 2025 [19,20,21,22,23,24,25,26,27]. The screening process is summarized in Figure 3, and baseline characteristics and main clinical findings are presented in Table 1.

### 3.2. Study Baseline Characteristics

The included studies were published between 2024 and 2026 and evaluated XAAI systems across a range of experimental and validation settings. Study designs varied, with several studies representing proof-of-concept implementations (e.g., CBM-RAG, ENTAgents, ArgMed-AgentN, DSM5AgentFlow, and BRAD) [20,21,24,25,27]. In contrast, others incorporated retrospective validation using benchmark or real-world datasets (e.g., ADAM-1, DrugAgent, EMAI, and CASSIA) [19,22,23,26]. Importantly, no studies reported prospective clinical deployment and should be interpreted as being at early stages of development, with varying proximity to clinical applicability.

Application domains spanned radiology, otolaryngology, neurology, psychiatry, and broader biomedical research tasks, reflecting both direct clinical use cases and upstream discovery or annotation workflows relevant to healthcare innovation. The primary clinical and biomedical tasks addressed included clinical decision support systems (CDSSs) [20,21,22,24,27], question answering (QA) [27], mental health screening and assessment [24], diagnostic and report generation or summarization [20,22,23], drug–target interaction prediction [19], biomarker discovery [25], and single-cell annotation [26]. Studies were conducted predominantly in China and the United States, with additional contributions from Germany and the Netherlands. Across domains, agentic systems were evaluated against single-agent large language model (LLM) baselines, traditional machine learning models, or non-reasoning pipelines, enabling comparative assessment of both performance and interpretability-related outcomes.

### 3.3. Overview of Agentic Systems

**Figure 4:**
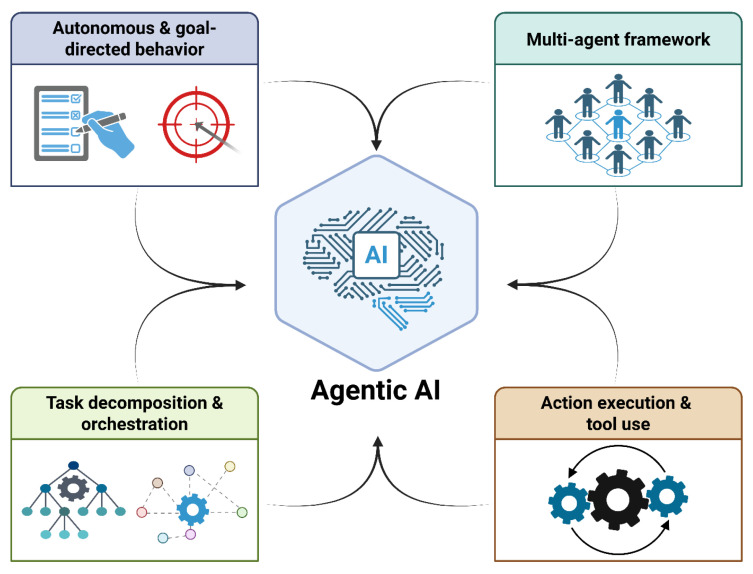
Core architectural components of XAAI in healthcare. Created in BioRender. Collaco, B. (2026) https://BioRender.com/3eyye5r (accessed on 12 February 2026).

Across the included studies, agentic AI systems demonstrated a diverse yet convergent set of architectural patterns. Although additional agentic characteristics were identified in individual studies, such as adaptability and memory [5,28], the heterogeneity of reporting and implementation made exhaustive enumeration impractical; therefore, we synthesized the most consistently reported and conceptually central features to provide a structured, comparable overview. Importantly, this also underscores that the included systems differed in the degree of agentic behavior, reflecting a spectrum of implementations with varying levels of autonomy, task decomposition, tool use, and coordination [1]. These features reflected a shared emphasis on autonomy, task decomposition, and structured decision-making (Figure 4).

Autonomy was operationalized through explicit planning, execution, and termination of tasks, including information retrieval, reasoning, verification, and report generation. Following an initial user input, systems executed multi-step reasoning or analytical workflows without requiring human intervention during inference. Even in systems employing a single agent (e.g., BRAD), autonomy was achieved through internal orchestration of tools and workflow stages rather than through agent–agent interaction [25].

Building upon this autonomous foundation, most systems adopted explicit multi-agent frameworks, defining multiple agents with distinct and complementary roles. These architectures contrasted with the single-agent, tool-augmented design observed in BRAD [25], where specialization was achieved via modular tools rather than separate agents. Multi-agent systems were predominantly role-based, with agents assigned to cognitively separable subtasks such as drafting, verification, retrieval, synthesis, supervision, or reporting [22,26,27].

Despite this shared multi-agent structure, coordination strategies varied across studies. Some systems employed hierarchical control, with a manager or coordinator agent governing execution order and termination, such as EMAI and DrugAgent [19,23]. Others relied on iterative or recursive interaction patterns, such as drafter–revisor–supervisor loops for ENTAgents [27], generator–verifier cycles for ArgMed-Agents [21], or annotator–validator refinement loops for CASSIA [26]. Dialogue-centric coordination was observed in DSM5AgentFlow, where the therapist and client agents interacted before the diagnostician agent performed diagnostic reasoning [24].

Additionally, explicit task decomposition was a consistent feature across systems, with complex clinical or biomedical objectives divided into logically distinct subtasks. For example, CBM-RAG separated disease-specific reasoning from report synthesis [20]; ADAM-1 distinguished computational analysis from narrative interpretation [22]; and CASSIA partitioned annotation, validation, quality scoring, and reporting into discrete stages [26]. Orchestration was generally sequential but adaptive, allowing for iterative refinement when uncertainty or low confidence was detected.

Tool use was universal. Agents interacted with external resources, including biomedical literature databases, clinical guidelines, knowledge graphs, enrichment platforms, statistical software, and machine-learning models. Retrieval-augmented generation (RAG) was frequently incorporated to ground reasoning in external evidence, particularly in systems aiming to mitigate hallucinations or enhance reliability, such as CBM-RAG, DrugAgent, DSM5AgentFlow, CASSIA, and BRAD [19,20,24,25,26].

Taken together, these studies illustrate a spectrum of agentic designs, ranging from single-agent, tool-orchestrating systems to deeply modular multi-agent frameworks incorporating iterative self-correction and quality control. Despite architectural variation, all systems shared core agentic properties, autonomy, goal-directed execution, task decomposition, and tool interaction, supporting their classification as explainable agentic AI systems within clinical and biomedical contexts [2,5,28].

### 3.4. Explainable Methods Used

Across included studies, explainability was predominantly implemented as intrinsic and workflow-native, with explanations generated during execution and less appended through standalone post hoc modules (Table 2). This pattern reflects the fact that all systems relied on multi-agent workflows in which intermediate reasoning states, role-specific outputs, and verification steps were naturally surfaced as user-facing rationales. As a result, explanations were embedded within the agentic process itself rather than layered externally after inference [21,24,27].

In terms of explanation form, textual explanations were universal. These typically consisted of stepwise reasoning traces, structured narratives, or clearly delineated outputs from specialized agent roles. In addition, most systems incorporated example-based grounding, operationalized as explicit links to retrieved clinical guidelines, biomedical literature, databases, ontologies, or knowledge graph path [19,25,26]. Such grounding mechanisms were especially prevalent in systems that integrated retrieval into their inference pipelines, thereby supporting traceability and reducing hallucination risk.

In practice, intrinsic explainability was most commonly operationalized through textual and example-based explanation forms, whereas feature- or concept-based explanations were primarily associated with post hoc methods. These were comparatively uncommon and appeared only in systems that embedded structured predictive components amenable to attribution, including saliency and concept bottleneck representations in CBM-RAG and SHAP-based feature importance in ADAM-1. Consistent with this distribution, post hoc explainability was not used as a standalone strategy but appeared exclusively in hybrid systems that combined intrinsic agent reasoning with attribution-based methods [20,22].

Notably, these hybrid approaches may more closely approximate what is traditionally considered mechanistic explainability, as they integrate process-level transparency with variable- or feature-level attribution. In this setting, intrinsic reasoning traces describe how decisions are constructed within the agentic workflow, while attribution-based methods provide insight into which inputs contributed to those decisions. When aligned, these complementary perspectives can enhance interpretability and offer partial verification of model behavior, bringing such systems closer to mechanistically informative explanations than purely reasoning-based approaches.

By contrast, systems such as DSM5AgentFlow, although providing structured, evidence-grounded rationales linking patient responses to DSM-5 criteria, primarily generate reasoning narratives that are not formally validated against the underlying decision process and should therefore be interpreted as process-level interpretability [24]. However, the degree of alignment between reasoning outputs and underlying decision mechanisms remains uncertain and requires further validation.

Regarding scope, most studies provided only local explanations, meaning they justified individual cases, queries, or decisions and did not characterize the overall system decision logic. Only two systems supported both local and global explanation layers: ADAM-1 combined instance-level rationales with population-level feature-attribution summaries [22], and EMAI produced case-level explanations alongside generalized “experience” rules induced from historical report clusters [23]. The relative scarcity of global explainability layers may suggest that current XAAI implementations prioritize runtime transparency over aggregate system-level characterization.

A consistent alignment between application type and explanation form was also observed. Systems focused on CDSSs, screening, and biomedical reasoning (e.g., ENTAgents, DrugAgent, DSM5AgentFlow, BRAD, and CASSIA) primarily relied on a “reasoning + evidence” paradigm, combining textual rationales with explicit grounding in retrieved sources [19,24,25,26,27]. In contrast, feature-/concept-based explanations appeared only in studies that intentionally incorporated explainable tools into the pipeline (e.g., post hoc explainability). ArgMed-Agents was methodologically distinctive in its structured argumentation framework; however, its user-facing explanations remained textual, presented as organized reasoning traces rather than attribution maps or feature weights [21].

In summary, the current XAAI landscape in healthcare appears to be centered on runtime textual justification strengthened by evidence grounding. At the same time, global explainability and attribution-based transparency remain comparatively limited and largely confined to hybrid designs. Importantly, given the modest number of eligible studies and the early stage of this research area, these patterns should be interpreted cautiously as indicative of emerging design tendencies. In addition, although transparency and interpretability were frequently described through reasoning traces and evidence grounding, explicit mechanisms for human oversight, clinician validation, and intervention points were not consistently reported across studies, limiting systematic comparison of how such oversight is operationalized in current XAAI systems.

### 3.5. Main Clinical Findings

Across application domains, reported outcomes of XAAI systems can be broadly categorized into performance improvements, interpretability-related benefits, and trust or usability-related effects. Overall, studies reported generally favorable trends across these domains relative to single-agent or non-reasoning baselines, though these findings were based on heterogeneous evaluation frameworks.

In radiology, systems such as CBM-RAG and EMAI were reported to demonstrate performance improvements through enhanced classification and report summarization accuracy. In parallel, interpretability benefits were achieved by linking visual regions to clinically meaningful concepts and by generating evidence-grounded reports, yielding structured, human-interpretable outputs. These systems also reported improvements in trust-related metrics, including reduced hallucinations and increased perceived reliability of generated reports [20,23].

For CDSSs and specialty QA, multi-agent workflows such as ENTAgents demonstrated performance gains over baseline LLM approaches, particularly through self-correction mechanisms. Interpretability-related benefits were reflected in clearer and more comprehensive reasoning narratives [21]. Similarly, ArgMed-Agents improved performance relative to direct generation and CoT baselines while enhancing interpretability by making reasoning processes more explicit, particularly in clarifying why specific clinical options were selected or rejected [27].

In neurology, particularly for Alzheimer’s disease detection and reporting, ADAM-1 achieved higher and more stable F1 scores than traditional machine learning models. Interpretability was supported by integrating intrinsic reasoning and post hoc feature attribution, producing decision-linked explanatory outputs [22]. In psychiatry, DSM5AgentFlow reported F1 scores up to 77%, while interpretability-related benefits included structured, criteria-based reasoning. Trust and usability-related claims were reflected in the generation of transparent and auditable diagnostic assessments, although these were not formally evaluated in user-centered studies [24].

In biomedical applications, including drug–target interaction prediction (DrugAgent), biomarker discovery (BRAD), and single-cell annotation (CASSIA), agentic pipelines demonstrated performance gains in predictive and analytical tasks. Interpretability-related benefits were observed through the integration of structured evidence sources, such as knowledge graphs, literature retrieval, and citation logs, enabling traceable reasoning processes. In CASSIA, multi-agent reasoning combined with confidence and consensus mechanisms improved annotation accuracy by 12–41% across large-scale cell populations. Trust-related features, such as confidence scoring and auditability of reasoning steps, were also reported, although their impact on real-world decision-making remains to be established [19,25,26].

## 4. Discussion

This scoping review maps an emerging body of work on XAAI in healthcare and related domains, identifying both convergent architectural patterns and important gaps in evaluation and reporting, with a focus on explainability as an operational property. Across the included studies, agentic systems were reported to demonstrate autonomy, goal-directed execution, explicit task decomposition, and tool use. Most adopted multi-agent frameworks assigned specialized roles, such as drafting, verification, retrieval, synthesis, and reporting, and coordinated them through iterative refinement or hierarchical control. These architectural features are broadly consistent with descriptions of agentic AI as systems that pursue complex goals through planning, tool invocation, and adaptive multi-step workflows [29].

These same architectural characteristics help explain why explainability in XAAI is typically embedded within the workflow itself rather than implemented as a separate post hoc interpretability layer, a central finding of this review. In contrast to traditional AI systems, where explanations are often appended after inference, explanations in XAAI are generated during execution as part of the agentic process. The prevalence of multi-agent workflows naturally produces intermediate reasoning states, role-specific outputs, verification steps, and tool traces that can be surfaced as user-facing rationales [30].

In practical terms, XAAI in healthcare appears to be process-explainable first: clinicians can inspect how an output was produced, who performed which subtask, in what order, and with which tools, even when the internal model parameters remain opaque. Importantly, although agentic systems often expose structured reasoning trajectories, these should not be assumed to represent causally faithful decision-making processes, as such explanations may reflect plausible or post hoc rationalizations rather than the true mechanisms underlying model outputs.

The form that this intrinsic explainability takes further clarifies the current landscape. Textual explanations were consistently reported across included systems, typically presented as step-by-step reasoning traces, structured narratives, or explicitly labeled outputs from distinct agent roles. In addition, example-based grounding, operationalized through explicit links to retrieved literature, clinical guidelines, database entries, or knowledge graph paths, was also common. The widespread integration of retrieval mechanisms likely reflects an effort to enhance traceability and mitigate hallucinations within agentic pipelines. By contrast, feature- or concept-based explanations appeared only in systems incorporating conventional predictive components amenable to attribution, such as saliency maps, concept bottleneck representations, or SHAP analyses over structured variables [31,32,33]. These systems were the only ones classified as hybrid (intrinsic plus post hoc), whereas the remainder relied exclusively on intrinsic mechanisms. Collectively, this distribution suggests that, in the current literature, explainability in agentic healthcare systems is predominantly delivered as narrative justification and evidence citation rather than as faithful attribution of internal model variables. In other words, XAAI is currently described mainly in terms of form (what is shown) and process (how the system proceeded), while deeper mechanistic transparency remains uncommon unless explicitly engineered through structured predictive models.

Closely related to the explanation form is the issue of scope. Most systems were reported to support local explanations, providing rationales for individual cases, queries, or outputs. Only a small subset offered both local and global explanation layers, typically by combining case-level reasoning with population-level summaries such as aggregate feature importance or generalized experience rules derived from historical clusters. This imbalance is noteworthy because global explainability, understanding how a system behaves across cases, is critical for governance, calibration, bias detection, and safe deployment [34,35]. Reliance on local explanations alone may limit the ability to detect systematic failure modes, brittle tool dependencies, or performance drift over time.

When situated within the broader XAI literature, the explainability profile of XAAI appears to differ in several respects from that of traditional, non-agentic healthcare AI. In conventional clinical machine learning, explainability has often centered on post hoc feature-attribution methods applied to static predictive models trained on imaging or structured electronic health record data [31,36]. These approaches typically emphasize identifying influential inputs and have been associated with ongoing concerns regarding faithfulness, stability, and interpretability [37,38,39]. In contrast, agentic systems consist of orchestrated workflows combining language models, tools, retrieval modules, and verification loops. Consequently, explainability may shift from feature attribution toward narrative reasoning traces, evidence grounding, and tool provenance. While this form of process-level transparency may align with aspects of clinical reasoning, it does not necessarily ensure that explanations are causally tied to underlying decision mechanisms. Accordingly, evaluation paradigms developed for classical XAI may not fully translate to agentic systems. Instead, XAAI may benefit from complementary assessment approaches focused on workflow fidelity, such as verifying whether cited evidence and tool outputs meaningfully contribute to decisions, alongside conventional measures of performance and interpretability [40].

Importantly, agentic design alone does not guarantee explainability. Beyond the systems included in this review, a growing number of healthcare agentic frameworks emphasize autonomy, compliance, or planning capabilities without explicitly engineering runtime explanation mechanisms. For example, the Compliance Agentic Model (CAM) proposed by Menezes et al. (2025) conceptualizes transparency primarily at the governance level, through auditability, policy enforcement, and supervisory monitoring, rather than as structured, case-specific explanations embedded within clinical workflows [41]. While exposing intermediate reasoning steps or retrieved evidence may create the appearance of transparency, it does not necessarily ensure that explanations are well-defined, verifiable, or clinically interpretable.

Similarly, GPT-Plan (Wang et al., 2025) operationalizes multi-step clinical reasoning through autonomous planning and tool invocation [42], and ChatExosome (Yang, 2025) integrates deep learning with retrieval-augmented language modeling to support hepatocellular carcinoma diagnosis [43]. Although both systems demonstrate clear agentic properties, explainability is primarily conveyed through conversational outputs, planning traces, or cited sources rather than through explicitly defined and systematically evaluated explanation mechanisms. Taken together, these examples illustrate that transparency at the level of governance or reasoning transcripts does not inherently translate into structured, clinically actionable explainability. This distinction underscores a critical point: explainable agentic AI requires deliberate architectural integration of runtime explanation mechanisms, rather than assuming that autonomy, regulatory alignment, or access to reasoning artifacts alone will yield meaningful interpretability in safety-critical clinical contexts.

**Figure 5:**
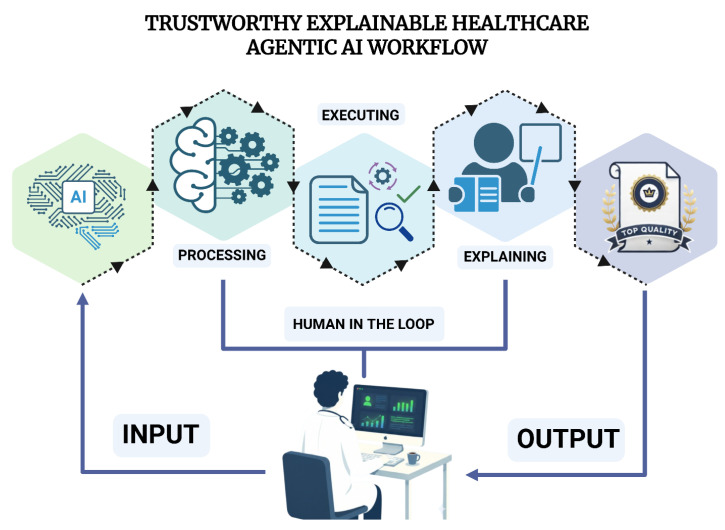
Trustworthy explainable healthcare agentic AI workflow. Created in BioRender. Collaco, B. (2026) https://BioRender.com/3eyye5r (accessed on 12 February 2026).

In this regard, the apparent predominance of intrinsic, workflow-native explainability aligns with international regulatory and governance frameworks (e.g., the EU AI Act and FDA framework) that emphasize transparency, traceability, documentation, and human oversight in healthcare AI [44,45,46]. However, explicit mechanisms for structured human-in-the-loop oversight were rarely described in the included studies, as reported in the qualitative synthesis of the included studies’ system designs, and none clearly operationalized how clinicians or domain experts would supervise, validate, or intervene in autonomous, agentic workflows. This gap is particularly notable given that regulatory guidance consistently frames human oversight not merely as a design principle but as an enforceable safety requirement in high-risk AI systems [47,48], and as a foundational condition for achieving trustworthy agentic AI in clinical settings (Figure 5). The nature of workflow-native explainability itself introduces additional safety considerations. Textual rationales and retrieved evidence can enhance transparency; however, they may also introduce new failure modes, including plausible but incorrect reasoning narratives, weakly linked citations, or incomplete provenance between tool outputs and final recommendations [37,49,50]. These risks underscore the need for verifiable explainability, in which citations are linked to specific retrieved passages, tool outputs are deterministically preserved and referenced, intermediate agent states are logged with dependencies, and systems can surface uncertainty, disagreement, or conflicting evidence when present. Encouragingly, several included studies incorporate partial safeguards, such as verifier agents, confidence scoring, and explicit evidence tagging, indicating an emerging pathway toward more robust oversight mechanisms.

Despite these insights, the conclusions of this review must be interpreted in light of several limitations. The number of studies included remains modest, reflecting the field’s early stage, and most systems were evaluated in experimental or proof-of-concept settings rather than in prospective clinical deployments. Furthermore, substantial heterogeneity in tasks, architectures, datasets, and reporting practices limits direct comparison across systems. In addition, reported outcomes related to performance, interpretability, transparency, and trustworthiness were extracted as described by study authors and were not independently verified or standardized, limiting their direct comparability across studies. The observed dominance of textual and example-based intrinsic explainability should therefore be considered a snapshot of a rapidly evolving literature rather than a definitive characterization of mature clinical implementations.

Moreover, while it is necessary to capture recent developments in this rapidly evolving field, the inclusion of preprints and non-peer-reviewed sources introduces potential variability in methodological rigor and reporting quality. Additionally, the explainability taxonomy applied in this review, while grounded in established XAI frameworks, was adapted to XAAI and has not undergone formal validation; therefore, it should not be interpreted as a definitive framework. Finally, the eligibility criteria requiring explicit reporting of explainability features may have excluded agentic AI systems with implicit or underreported explainability, potentially introducing a selection bias toward systems already framed as explainable.

Within these constraints, several directions for future research become evident. First, standardized evaluation of explanations is urgently needed, as most studies emphasize performance gains and qualitative interpretability claims without systematically assessing explanation fidelity, clinician comprehension, or impact on decision quality [51,52,53]. In parallel, future work should aim to refine and validate explainability taxonomies tailored to agentic AI systems, to support more consistent evaluation and comparison across studies. Second, global explainability and monitoring capabilities should be strengthened to support governance and post-deployment oversight.

Third, hybrid approaches that combine workflow-level transparency with mechanistic model-level explanations (when structured predictive components are present) may help bridge process explainability with variable-level understanding. In particular, future research should distinguish between plausibility and causal faithfulness of explanations, ensuring that reasoning trajectories accurately reflect the mechanisms underlying model outputs.

Finally, human factors and workflow integration deserve deeper investigation. Explanations must not only exist but also be usable, align with clinical reasoning practices, support efficient verification under time constraints, facilitate meaningful human supervision, and avoid cognitive overload from multi-agent outputs [53]. Systematic evaluation of how clinicians interact with agentic explanations, including override behavior, trust calibration, and error detection, will be critical for translating XAAI from experimental prototypes to safely governed clinical systems.

## 5. Conclusions

This scoping review demonstrates that XAAI in healthcare is an emerging but rapidly evolving field characterized by multi-agent, tool-augmented systems that can integrate explainability directly into their execution workflows. Across included studies, explainability was predominantly intrinsic and local, delivered through textual rationales frequently grounded in external evidence. At the same time, feature- or concept-based and global explanation mechanisms were comparatively rare and largely confined to hybrid architectures. These findings suggest that current XAAI systems tend to prioritize process transparency and evidence traceability over mechanistic model-level attribution. Although promising, the evidence base remains limited and largely experimental, underscoring the need for standardized evaluation frameworks, clearer reporting of agentic and explanatory capabilities, and stronger validation in real-world clinical settings. Advancing XAAI will require balancing autonomous multi-agent reasoning with robust, verifiable, and clinically meaningful explanations to ensure safe, trustworthy deployment in healthcare environments.

## Figures and Tables

**Table 1:** Baseline characteristics of included studies.

**Study, Year**	**Country**	**Study Design**	**XAAI**				
			**Name**	**Domain**	**Main Clinical Task**	**Key Agentic Features**	**Included Agents**
Alam 2025 [20]	Germany	Proof-of-concept	CBM-RAG	Radiology, CDSS	Chest X-ray disease classification and report generation	Autonomy Multi-agent framework Task decomposition and orchestration Tool use	Pneumonia Agent COVID-19 Agent Normal Agent Radiologist Agent Report Writer Agent
Chan 2025 [27]	China	Proof-of-concept	ENTAgents	Otolaryngology, CDSS	QA	Autonomy Multi-agent framework Task decomposition and orchestration Tool use	Drafter Agent Revisor Agent Supervisor Agent Search Agents
Hong 2024 [21]	China	Proof-of-concept	ArgMed-AgentN	CDSS	Clinical decision reasoning (treatment/medication decisions)	Autonomy Multi-agent framework Task decomposition and orchestration Tool use	Generator Agent(s) Verifier Agent Reasoner Agent
Huang 2025 [22]	China	Retrospective validation	ADAM-1	Neurology, CDSS	Alzheimer’s disease detection/classification + reporting	Autonomy Multi-agent framework Task decomposition and orchestration Tool use	Computational Agent Summarization Agent Classification Agent
Inoue 2025 [19]	USA	Retrospective validation	DrugAgent	Biomedicine	Drug–target interaction prediction	Autonomy Multi-agent framework Task decomposition and orchestration Tool use	Coordinator Agent AI Agent KG Agent Search Agent Reasoning Agent
Li 2026 [23]	China	Retrospective validation	EMAI	Radiology	Report summarization	Autonomy Multi-agent framework Task decomposition and orchestration Tool use	Manager Agent Experience Retrieval Agent Report Retrieval Agent Findings Analysis Agent Impression Composition Agent
Ozgun 2025 [24]	Netherlands	Proof-of-concept	DSM5AgentFlow	Psychiatry, CDSS	Mental health screening and diagnosis	Autonomy Multi-agent framework Task decomposition and orchestration Tool use	Therapist Agent Client Agent Diagnostician Agent
Pickard 2025 [25]	USA	Proof-of-concept	BRAD	Biomedicine	Automated biomarker discovery, enrichment analysis, and report generation	Autonomy Task decomposition and orchestration Tool use	BRAD Agent
Xie 2024 [26]	USA	Retrospective validation	CASSIA	Biomedicine	Cell type annotation of single-cell RNA-sequencing data	Autonomy Multi-agent framework Task decomposition and orchestration Tool use	Annotator Agent Validator Agent Formatting Agent Quality Scoring Agent Reporter Agent

The table summarizes country of origin, study design, system name, clinical or biomedical domain, primary task, core agentic properties (autonomy, task decomposition, orchestration, tool use, and multi-agent structure), and the composition of agents within each framework. CDSS = clinical decision support system; QA = question-answering; KG = knowledge graph.

**Table 2:** Explainability characteristics of included XAAI systems in healthcare.

Study, Year	Scope	Specificity	Type	Approach	Explanation Form	Main Clinical Findings
Alam 2025 [20]	Local	Model-specific	Intrinsic + Post hoc	CBM-based classification Multi-agent RAG workflow Saliency-based attribution	**Feature-based:** Clinical concepts, contribution scores, saliency heatmaps **Textual:** Natural-language reports, CoT, ReAct reasoning **Example-based:** Retrieved clinical guidelines	**Performance:** Improved classification and report generation **Interpretability:** Concept-linked visual and textual explanations **Trust/Usability:** Reduced hallucinations; improved transparency
Chan 2025 [27]	Local	Model-specific	Intrinsic	Multi-agent LLM CDSS RAG + role-based workflow CoT reasoning	**Textual:** CoT reasoning, stepwise agent outputs **Example-based:** Retrieved clinical guidelines or contextual cases	**Performance:** Improved QA accuracy (e.g., +11.3%) **Interpretability:** Clearer, structured reasoning **Trust/Usability:** Self-correction improved transparency
Hong 2024 [21]	Local	Model-specific	Intrinsic	Multi-agent argumentation framework Argumentation graph + symbolic solver Iterative self-argumentation	**Textual:** structured argumentation steps, critical questions, explicit reasoning traces, competing clinical decisions and counter-arguments	**Performance:** Higher accuracy vs. CoT/direct generation **Interpretability:** Explicit reasoning and alternative comparison **Trust/Usability:** Improved decision transparency
Huang 2025 [22]	Local + Global	Mixed (specific and agnostic)	Intrinsic + Post-hoc	Multi-agent reasoning framework (microbiome + clinical data) RAG + CoT reasoning SHAP-enhanced XGBoost integration	**Feature-based:** SHAP importance, diversity metrics **Textual:** structured CoT summaries and classification rationales **Example-based:** retrieved biomedical evidence	**Performance:** Higher and more stable F1 vs. XGBoost **Interpretability:** Combined feature attribution + reasoning **Trust/Usability:** Improved decision traceability
Inoue 2025 [19]	Local	Model-specific	Intrinsic	Multi-agent LLM-based framework for drug–target prediction ML (DeepPurpose) + KG + literature integration Coordinator-based orchestration	**Textual:** step-by-step reasoning traces, agent-specific rationales, CoT and ReAct explanations **Example-based:** explicit KG paths and literature-derived evidence	**Performance:** Achieved 45% improvement F1 vs. non-reasoning baseline **Interpretability:** Integrated ML + KG + literature explanations **Trust/Usability:** Improved transparency and reliability
Li 2026 [23]	Local + Global	Model-specific	Intrinsic	Multi-agent workflow for report summarization Experience-guided rule induction Case-based retrieval	**Textual:** sub-finding analyses + correlation rationales + induced experience rules **Example-based**: retrieved similar radiology reports	**Performance:** Improved summarization accuracy **Interpretability:** Rule-based and case-linked explanations **Trust/Usability:** Improved clinician trust and error detection
Ozgun 2025 [24]	Local	Model-specific	Intrinsic	Multi-agent DSM-5 diagnostic workflow Therapist–client simulation RAG-grounded reasoning	**Textual:** step-by-step diagnostic rationales, criterion-linked reasoning, tagged evidence **Example-based:** retrieved DSM-5 passages + diagnostic evidence	**Performance:** Achieved up to 77% F1 **Interpretability:** Criterion-linked diagnostic reasoning **Trust/Usability:** Transparent, auditable assessments
Pickard 2025 [25]	Local	Model-specific	Intrinsic	Agentic bioinformatics workflow LLM + external tools integration Full logging and orchestration	**Textual:** CoT interpretations, stepwise reasoning narratives, tool-selection rationales **Example-based:** retrieved literature excerpts, database citations	**Performance:** Improved biomarker workflow accuracy **Interpretability:** Traceable, reproducible reasoning **Trust/Usability:** Enhanced transparency and reproducibility
Xie 2024 [26]	Local	Model-specific	Intrinsic	Multi-agent LLM framework for RNA-seq annotation Modular agent workflow Optional RAG + validation agents	**Textual:** structured step-by-step reasoning, validation narratives, quality and confidence reports **Example-based:** retrieved marker genes, ontology hierarchies, database-supported evidence	**Performance:** Improved annotation accuracy 12–41% **Interpretability:** Transparent, auditable reasoning **Trust/Usability:** Confidence scoring + reduced hallucinations

The table summarizes the scope (local vs. global), model specificity, explainability type (intrinsic vs. post hoc), implementation approach, explanation form (textual, feature-/concept-based, example-based), and reported clinical or biomedical findings for each system. CBM = Concept Bottleneck Model; RAG = Retrieval-Augmented Generation; LLM = Large Language Model; CoT = Chain-of-Thought; ML = machine learning; KG = knowledge graph; CDSS = clinical decision support system.

## Data Availability

The data supporting the findings of this review are available within the article and its Supplementary Materials. Extracted information from the included studies is summarized in the main text and tables.

## Supplementary Materials

The following supporting information can be downloaded at https://www.mdpi.com/article/10.3390/bioengineering13050513/s1. Supplementary Document S1: Preferred Reporting Items for Systematic reviews and Meta-Analyses extension for Scoping Reviews (PRISMA-ScR) Checklist; Supplementary Document S2: Full Search Strategy Conducted on 24 November 2025.
