# Supplementary material for: Explainable Agentic Artificial Intelligence in Healthcare: A Scoping Review
Source: Bioengineering (Basel). 2026 Apr 28;13(5):513. doi: 10.3390/bioengineering13050513 (PMC13203805; doi:10.3390/bioengineering13050513)
Supplement: Supplementary file 1 [file bioengineering-13-00513-s001.zip › bioengineering-4247861 Supplementary Document S2.pdf]

bioengineering

MDPI

Review

Explainable Agentic Artificial Intelligence in Healthcare: A Scoping Review

Bernardo G. Collaco [1], Srinivasagam Prabha [1], Cesar A. Gomez-Cabello [1], Syed Ali Haider [1], Ariana Genovese [1], Nadia G. Wood [2], Narayanan Gopala [3], Raghunath Raman [3], Erik O. Hester [3], Antonio Jorge Forte [1,3,4,*]

[1] Division of Plastic Surgery, Mayo Clinic, 4500 San Pablo Rd S, Jacksonville, FL 32224, USA
[2] Department of Radiology AI IT, Mayo Clinic, Rochester, MN 55905, USA
[3] Center for Digital Health, Mayo Clinic, Rochester, MN 55905, USA
[4] Department of Artificial Intelligence and Informatics, Mayo Clinic, Jacksonville, FL 32224, USA
* Correspondence: ajvforte@yahoo.com.br

Supplementary Material

Full Search Strategy (Nov 24 2025)

PUBMED > (agentic OR "AI agent" OR "autonomous agent" OR "multi-agent") AND (explainable OR XAI OR XAAI OR explainability OR interpretability OR interpretable OR transparent OR transparency OR interpretability OR "Transparency"[Mesh]) AND ("health care" OR healthcare OR medical OR clinical OR medicine OR "clinical decision" OR diagnosis OR intervention OR management OR patient OR "patient education" OR "Clinical Medicine"[Mesh] OR "Decision Making, Computer-Assisted"[Mesh]) >> 251

EMBASE > ('agentic' OR 'ai agent' OR 'autonomous agent' OR 'multi-agent') AND ('explainable' OR 'xai' OR 'xaai' OR 'explainability' OR 'interpretable' OR 'transparent' OR 'transparency' OR 'interpretability' OR 'transparency'/exp) AND ('health care' OR 'healthcare' OR 'medical' OR 'clinical' OR 'medicine' OR 'clinical decision' OR 'diagnosis' OR 'intervention' OR 'management' OR 'patient' OR 'patient education' OR 'clinical medicine'/exp OR 'decision support system'/exp) >> 94

IEEE XPLORE> (agentic OR "AI agent" OR "autonomous agent" OR "multi-agent") AND (explainable OR XAI OR XAAI OR explainability OR interpretability OR interpretable OR transparent OR transparency OR interpretability) AND ("health care" OR healthcare OR medical OR clinical OR medicine OR "clinical decision" OR diagnosis OR intervention OR management OR patient OR "patient education") >> 361

ACM Digital Library > (agentic OR "AI agent" OR "autonomous agent" OR "multi-agent") AND (explainable OR XAI OR XAAI OR explainability OR interpretability OR interpretable OR transparent OR transparency OR interpretability) AND ("health care" OR healthcare OR medical OR clinical OR medicine OR "clinical decision" OR diagnosis OR intervention OR management OR patient OR "patient education") >> 396
